# Towards an understanding of multimodal traits of female reproduction in chimpanzees

**DOI:** 10.1007/s10329-022-00995-1

**Published:** 2022-06-28

**Authors:** Marlen Kücklich, Susann Jänig, Lars Kulik, Claudia Birkemeyer, Brigitte M. Weiß, Anja Widdig

**Affiliations:** 1 Behavioural Ecology Research Group, Faculty of Life Sciences, University of Leipzig, Talstraße 33, 04103 Leipzig, Germany; 2 Primate Behavioural Ecology Research Group, Department of Human Behavior, Ecology and Culture, Max Planck Institute for Evolutionary Anthropology, Deutscher Platz 6, 04103 Leipzig, Germany; 3 Mass Spectrometry Research Group, Institute of Analytical Chemistry, University of Leipzig, Linnéstraße 3, 04103 Leipzig, Germany

**Keywords:** Olfactory communication, Olfaction, Gas chromatography—mass spectrometry, Chemical composition, Menstrual cycle, Anogenital swelling

## Abstract

Although primates have long been regarded as microsmatic, recent studies indicate that olfaction is an important sensory mode of primate communication, e.g., in the context of reproduction. However, large gaps remain in our understanding of primate olfactory traits, especially in the great apes. Female chimpanzees (*Pan troglodytes*) possess an exaggerated sexual swelling, which is an imprecise signal of fertility that is thought to serve to confuse paternity. However, some high-ranking males that copulate most frequently on the days when females are most fertile seem to have more precise information on the timing of ovulation, which suggests the existence of an olfactory fertility trait. In order to examine, and provide evidence for, fertility-related chemical information in female chimpanzees, we used gas chromatography—mass spectrometry to analyze the chemical composition of female body odor collected across the menstrual cycle during various stages of sexual swelling (97 samples of six females). The chemical composition was significantly affected by swelling stage, and eight substances were detected that were strongly related to the latter. The existence of an additional, olfactory, fertility trait may help males to fine-tune their sexual behavior or allow females to strengthen concealment of the exact timing of ovulation, and needs to be further investigated in follow-up studies. The results of our study provide much-needed evidence for the existence of an olfactory cue related to reproduction in chimpanzees, and form a basis for future studies on the interplay between visual and olfactory information on female fertility.

**Supplementary Information:**

The online version contains supplementary material available at 10.1007/s10329-022-00995-1.

## Introduction

Primates utilize various sensory modalities, including vision, acoustics and olfaction, for social communication, e.g., to convey information regarding kinship [olfaction in *Lemur catta* (Boulet et al. 2009); vision and acoustics in *Macaca mulatta* (Pfefferle et al. 2014a, b)] or dominance rank [vision in *Mandrillus leucophaeus* (Marty et al. 2009); acoustics in *Macaca nigra* (Neumann et al. 2010); olfaction in *Mandrillus sphinx* (Vaglio et al. 2016)]. Sensory modalities involved in the transfer of specific information may act either alone or as multimodal traits, whereby different modalities transmit either redundant or complementary information (Partan and Marler 1999). The fertile phase of female primates, e.g., is frequently indicated by a female’s behavior, such as approaching males or reducing aggression, by visual traits such as sexual swellings, acoustic traits such as advertisement or copulation calls, as well as olfactory traits such as scent-marking behavior, in various combinations [e.g., *Cebuella pygmaea* (Converse et al. 1995); *Microcebus murinus* (Buesching et al. 1998); *Papio ursinus* (Clarke et al. 2009); *Papio anubis* (Rigaill et al. 2013)].

Whereas visual and acoustic traits of female fertility have been relatively well studied across primates, studies on olfactory traits of fertility are comparatively scarce (Drea 2015). However, over the years, it has become evident that olfaction may also play an important role in the transmission of social information in primates (e.g., Smith and Bhatnagar 2004; Drea et al. 2013). Studies in several primate species indicate that females convey olfactory information regarding their reproductive state through whole body odor, vaginal secretions, urine and feces (Drea et al. 2015). Furthermore, olfactory inspection of the female anogenital region by males increases during the fertile period [*P. ursinus* (Clarke et al. 2009); *P. anubis* (Rigaill et al. 2013)], and males react with increased exploratory behavior and sexual activity [*Macaca mulatta* (Michael et al. 1971); *Galago crassicaudatus* (Clark 1982); *Saguinus oedipus* (Ziegler et al. 1993); *Callithrix jacchus* (Smith and Abbott 1998); *Macaca arctoides* (Cerda-Molina et al. 2006b)] as well as a rise in testosterone levels and activation of brain areas linked to sexual behavior [*C. jacchus* (Ziegler et al. 2005; Snowdon et al. 2006); *M. arctoides* (Cerda-Molina et al. 2006a)] when presented with vaginal secretions from fertile females.

Specific substances related to the menstrual cycle of females have recently been identified, and males were observed to distinguish between female odor from different phases of the cycle in *C. jacchus* [an aromatic hydrocarbon, a heterocyclic compound, an ester and an alcohol (Kücklich et al. 2019)]. Certain substances were also found to be fertility related and to sexually stimulate male Old World monkeys [fatty acids in *M. mulatta* (Michael et al. 1971)], although the results could not be reproduced in a subsequent study [*M. mulatta* (Goldfoot et al. 1976)]. These types of fertility indicators could constitute “signals”, i.e., traits that have specifically evolved to provide information on fertility, or “cues”, which constitute traits that have not evolved to deliver fertility-related information to conspecifics, e.g., being by-products of changes in hormone levels (Wyatt 2014).

Chimpanzees (*Pan troglodytes*) are prime candidates for the study of multimodal traits of fertility, as female chimpanzees have several traits that may be indicative of their reproductive state during their menstrual cycle, which is around 36 days long (Tutin 1979; Deschner et al. 2003; Emery and Whitten 2003). They make copulation calls, although these seem not to vary with fertility (Townsend et al. 2011), and show proceptive as well as mating resistance behavior that varies between their fertile and non-fertile periods (Stumpf and Boesch 2006). In addition, they have exaggerated sexual swellings, which vary in size across their cycle, although these are not a precise signal of fertility (Emery Thompson 2005). The phase of increased swelling lasts for approximately 10–12 days (Emery and Whitten 2003), and indicates approaching ovulation, which occurs during the period of maximal swelling (Deschner et al. 2004). However, maximal swelling persists for up to 4 days after ovulation (Deschner et al. 2004), and is thus only an imprecise signal of the latter. This imprecise information on the actual timing of ovulation allows females to confuse paternity and reduce the risk of infanticide by attracting a high number of mating partners (Wrangham 2002; Kappeler and van Schaik 2004; Stumpf and Boesch 2006). Males compete intensely with each other over their access to females (Goodall 1986; Muller 2002), which restricts their mating opportunities [priority of access model (Altmann 1962)]. The potential for males to monopolize females is further reduced since several females of a group will show exaggerated sexual swellings synchronously (Goodall 1986; Ostner et al. 2008). As males should focus their mating efforts on the days when females are most fertile in order to mate with as many fertile females as possible, they would benefit from using precise indicators of female fertility, if available. Indeed, male chimpanzees alter their sexual behavior when female anogenital swellings increase in size and throughout the period of maximal swelling (Klinkova et al. 2005), and mating seems to be correlated with the period of peak fertility of females (Deschner et al. 2004; Emery Thompson 2005). Alpha males, in particular, were found to copulate more frequently during the fertile period compared to the other days of the maximal swelling phase (Deschner et al. 2004). These observations suggest that high-ranking males may benefit from additional information, e.g., a multimodal cue of fertility. Rigaill et al. (2013) hypothesized that the visual signal in *P. anubis* could provide a first sign of fertility to all males within visual range, whereas high-ranking males that can approach females closely could potentially gather more precise information from an olfactory inspection of females with sexual swellings. Also, females could benefit from a more precise indicator of ovulation that is only sensed by selected, competitive males, to obtain the best genes and protection for their infants (Kappeler and van Schaik 2004).

Observations of increased copulation rates at the time of highest fertility in females suggest that male chimpanzees may indeed rely on olfactory traits in a sexual context (Emery Thompson and Wrangham 2008). Certain fatty acids that are thought to be associated with female fertility in rhesus macaques (*Macaca mulatta*) (Michael et al. 1971) were also found in the vaginal secretions of female chimpanzees, but it remains unclear if these vary during the menstrual cycle (Fox 1982; Matsumoto-Oda et al. 2003). Notably, fertility-related changes in the odor of female chimpanzees could occur in genital odor and/or in the scent of the whole body (hereafter “body odor”). Several other individual attributes have been detected in the body odor of chimpanzees and other great apes [e.g., indicating individual identity (Hepper and Wells 2010); arousal level (Klailova and Lee 2014); species identity and age (Jänig et al. 2019)]. Chimpanzees sniff other individuals (i.e., genitalia, but also other body parts), and males specifically have been observed to sniff their conspecifics more often than females do (Matsumoto-Oda et al. 2007; Jänig et al. 2018). Accordingly, we expect information about reproductive state to be provided by the body odor of female chimpanzees in general, and not only by their genital odor, which may constitute a multimodal trait of fertility in combination with the visual signal of fertility, i.e., the sexual swelling.

Given the lack of studies demonstrating the existence of olfactory fertility traits in chimpanzees, our overarching aim was to provide evidence, based on chemical analyses, that olfactory information exists that is related to reproduction. Chimpanzees live in large, multi-male/multi-female groups, which split up regularly into smaller parties of variable composition (Nishida 1968; Goodall 1986). Males preferably join parties which include females with maximal sexual swellings (Matsumoto-Oda 1999). If an olfactory fertility cue exists that is even more precise than the visual signal, this could potentially help males to specifically join parties with females close to ovulation. Cycle-dependent olfactory traits could be subtle changes that arise as a result of physiological changes, such as the breakdown of hormones involved in the menstrual cycle, e.g., luteinizing hormone, follicle-stimulating hormone, progesterone and estradiol (Nadler et al. 1985). Sexual swelling appears to be induced by a rise in the levels of estrogens, and detumescence is correlated with a rise in progesterone after ovulation (Graham et al. 1972). Hence, olfactory changes could occur as by-products of these hormonal changes that accordingly follow the timing of sexual swellings, and thus act as similarly imprecise fertility traits as the visual signal. Ovulation can be assumed to occur 1 day before the increase in the level of progesterone (Deschner et al. 2004), and is related to pre-ovulatory peaks of luteinizing hormone and follicle-stimulating hormone which are preceded by an estradiol peak mid-cycle (Nadler et al. 1985). Thus, a hormone-related olfactory change could also occur at the time of ovulation. If this were true, olfactory information on fertility would be more precise than that provided by the visual signal. On the other hand, an imprecise olfactory cue could strengthen concealment of the actual timing of ovulation, and thus support the visual signal and allow females to confuse paternity. A more precise olfactory cue, however, would allow high-ranking males in particular to monitor females with maximal swelling to pinpoint the exact time of ovulation. Either way, the composition of female body odor would be expected, at the very least, to vary between swelling stages.

In this study, we related the chemical profiles of body odor samples of regularly cycling female chimpanzees to their stage of sexual swelling. We investigated whether chemical composition varies synchronously with the visual signal, and predicted a change in the former across the sexual cycle. If this prediction were true, it would suggest that anogenital swelling and body odor potentially comprise multimodal traits of fertility. For the purposes of this study, we used data collected as part of a larger study on various traits of great ape body odor [species identity, age and sex (Jänig et al. 2019)]. However, at the time of data collection, we were not able to obtain hormone samples from females in a reliable manner that would have enabled us to determine the exact timing of ovulation. Hence, given the lack of hormonal data, we cannot show whether an olfactory cue exists that is a more precise indicator of fertility than the visual signal. Our results nonetheless provide information on whether olfaction is related to reproduction at all, and thus provide a foundation for future studies that include an investigation of hormones in this context.

## Materials and methods

### Subjects

We conducted our study on captive chimpanzees (*Pan troglodytes verus*) at the Wolfgang Köhler Primate Research Centre (WKPRC) at Leipzig Zoo, Germany, in 2013. We analyzed samples from six adult females (aged between 13 and 20 years) from two different social groups (for details on housing and group composition, see supplementary Online Resource), which had a regular menstrual cycle, i.e., did not receive contraceptives (for at least 12 months prior to the study) and were not lactating.

### Sexual swelling stages

The sexual swelling stage of each female was routinely recorded by the zookeepers and categorized as one of the following: flat, increasing in size, maximal tumescence, decreasing in size. Mean cycle length was 35.6 ± 9.1 days and thus within the ranges previously published for chimpanzees [captive (Nadler et al. 1985); wild (Tutin 1979; Wallis 1997; Emery Thompson 2005)]. On average, the observed females were detumescent for 16.7 ± 5.4 days, the swellings increased in size for 4.3 ± 5.5 days, remained at maximal tumescence for 12.3 ± 3.4 days, and decreased in size for 2.4 ± 1.5 days, similar to data reported for wild chimpanzees (Deschner et al. 2003).

### Sample collection and preparation

The examined individuals are well trained in participating in behavioral experiments by the WKPRC. Samples of skin odor were collected in the morning prior to behavioral experiments. For the purpose of our study, individuals were trained by positive reinforcement (using food items) to come to the grid of the cage to participate voluntarily in the sampling by presenting a self-selected body part. Clean Lilibe cotton swabs (60% cotton wool, 25% polyester microfiber, 15% polyester), heated for 30 min at 130 °C before use (Birkemeyer et al. 2016), were held with sterilized metal tweezers and rubbed repeatedly over the skin and fur for approximately 20 s (similar to Célérier et al. 2010; Stoffel et al. 2015). The cotton swabs were immediately placed in pre-cleaned (washed with methanol and diethyl-ether) 4-mL glass vials (Rotilabo) and stored at − 80 °C until gas chromatography—mass spectrometry (GC–MS) analysis.

Given that the individual chimpanzees had full control over which body part they presented, the samples included odor from the arms, belly, back and legs, but not the genitals, as these were not readily presented by the animals. Hence, we sampled body parts that have the same types of apocrine and eccrine sweat glands (Ellis and Montagna 1962; Montagna and Yun 1963) representing general body odor rather than genital odor. Body and genital odor should be similarly affected by hormonal changes across the menstrual cycle, as previously shown in humans (Michael et al. 1974, 1975; Gildersleeve et al. 2012). Furthermore, behavioral observations of both chimpanzee groups at the WKPRC revealed that when males sniff females they focus on the genitals in just over half of cases and other body parts in the remaining cases (Jänig et al., in review). We controlled for the different body parts in our statistical analysis. Samples were not collected from the mouth, hands or feet to reduce contamination with odor from food or the environment. Furthermore, diet was likely to have only a minor impact on body odor variance within and between individuals, since animals were fed daily with similar food.

In total, 97 samples [16.2 ± 2.3 samples per individual female covering from five to eight (mean 7.2) menstrual cycles each)] were collected from the animals (for details see supplementary Online Resource Table 1). In addition, 42 control samples (pure cotton swabs not rubbed over the skin or fur but otherwise handled like the animal samples; one per sampling day) were collected to identify chemical substances that did not originate from the chimpanzees.

### Chemical analysis

We extracted the chemical substances for GC–MS analysis by adding 1.2 mL of* n*-hexane (Sigma Aldrich, Steinheim, Germany) to the cotton swab in a glass vial (Birkemeyer et al. 2016). The extract was concentrated stepwise to a volume of circa 60 µL, and 4 µL of this solution was injected into the GC (HP6890 Series GC System, Agilent, Waldbronn, Germany, with a HP5973 MSD Mass Selective Detector in electron-impact ionization mode at 70 eV) using splitless injection, for 2 min. The GC was equipped with a J&W Fisher DB35-MS column (30 m length, 0.25 mm inner diameter, 0.25 µm film; Agilent), with the inlet temperature set to 250 °C. Helium was used as the carrier gas at a flow rate of 1.7 mL/min. The temperature program ran for 40.5 min, starting at 35 °C for 2 min, followed by a heating step of 10 °C/min until 320 °C, which was held for 10 min. The solvent delay was set to 7 min. The ion source operated at 250 °C and the scan range was set to *m/z* 50–550.

### Data processing

From the GC–MS data we identified peaks, their retention times (RTs) and areas (intensity) using AMDIS v. 2.65 (Stein 1999). To determine substances that were repeatedly detected in our samples, we grouped consecutive RTs to one RT range (see also Weiß et al. 2018) using a self-written script performed in R version 3.2.3 (R Core Team 2015). We verified by manual inspection that mass spectral patterns of the peaks within a given RT range were consistent, i.e., each RT range was assumed to reflect one substance, resulting in 152 RT ranges. In the following, we thus use the term “substance” rather than “RT range.” At that stage of the study, the substances had not yet been chemically identified by using a library search, and were described only by their RT and specific patterns of signals with certain mass-to-charge-ratios (*m/z*).

We used a non-targeted approach to find substances that might be related to female fertility. We excluded 15 substances from the statistical analysis which had the same or a higher abundance in the control than in the animal samples (i.e., data on 137 of the 152 substances remained for analysis). For all the substances which were found to be potentially related to female fertility according to the statistical analysis, mass spectral comparisons using the National Institute of Standards and Technologies (NIST) Mass Spectral Library (NIST 14 software; NIST, Gaithersburg, MD) were conducted for their tentative identification. We report the best library hit per substance when (1) the top library hit was consistent over samples, (2) the match had a probability > 80 (NIST 14 value), and (3) the tentatively identified compound could be reasonably expected to elute at the given RT. Alternatively, where no library hit allowed tentative identification, we propose a structural classification based on interpretation of the spectrum. The mass spectra of the substances discussed in this article are provided in supplementary Online Resource Fig. 2.

### Statistical analysis

First, we conducted an analysis of similarities (ANOSIM) to test whether chemical profiles of females at the same swelling stage were more similar to each other than profiles of females at different swelling stages. Following common practice (e.g., Stoffel et al. 2015), the ANOSIM was based on Bray–Curtis indices calculated from the log-transformed, standardized intensities of substances (intensity of substance divided by the summed intensity of all 137 substances × 100) for each combination of sample dyads. To control for repeated measurements per female, we used a customized ANOSIM (R script written by LK) that computed* P*-values by permuting swelling stages within individual females only. The α-level was set to 0.05 for all statistical analyses.

Second, we implemented two two-tailed generalized linear mixed models in R v. 3.2.3 (R Core Team 2015) using the package lme4 v. 1.1.11 (Bates et al. 2015) to determine if specific substances are associated with a particular swelling stage. Chemical datasets of animals may present several analytical challenges, such as (1) substances of potential biological relevance may have very high, or very low intensities; (2) the total intensities of samples can change due to environmental factors; and (3) the intensity of certain substances (e.g., those considered contaminants, such as plasticizers) can vary strongly, and thus affect the relative composition of odor profiles (van den Berg et al. 2006). To overcome these problems, we fitted the two models using two different responses:* standardized* (intensity of substance divided by the summed intensity of all 137 substances per sample × 100) and* transformed* [arcsine and log(*x* + 0.01)] peak areas; *standardized* peak areas* centered* (to a mean of 0) and* scaled* (to a SD of 1). In both cases, standardization was used to correct for changing total sample intensities. In the first approach, transformation was applied to achieve a normal distribution and to reduce the relative impact of large substances, while differences in the relative abundances of the various substances were maintained. In the second approach, centering and scaling was applied to adjust all the substances to the same size and to give all of them equal weight in the analyses (van den Berg et al. 2006). The responses were vectorized from a multivariate data matrix (Jamil et al. 2013) of samples (*n* = 97) and substances (*n* = 137). Swelling stage (dummy coded and centered) was fitted as fixed effect test predictor, and age (*z*-transformed) of the females was fitted as control predictor. Sample number and substance identity (ID) were included as random effects to prevent pseudo-replication as well as heteroscedastic variance due to the vectorized data matrix (Jamil et al. 2013). Other random effects were the day of observation, the sampled body part, as well as ID and group of the female. We included the random slope of the fixed effects test predictor (swelling stage) within substance as the actual test predictor [for detailed information, see Weiß et al. (2018)]. Additionally, swelling stage was fitted as a random slope within ID of the female to achieve more accurate estimates of the test predictor.

The check for normal distribution and homogeneity of the residuals by inspection of a* qq*-plot suggested no violation of assumptions. Plotting the residuals against fitted values revealed a slight bottom effect, but model stability indicated no influential cases. We determined variance inflation factors (VIF) to check for potential collinearity (Quinn and Keough 2002; Field 2005) calculated with the function vif of the package car (Fox and Weisberg 2011) and found no indication of collinearity (VIF_max_ = 1.01).

We compared the full model to a null model excluding the random slope within substance by using a likelihood ratio test (LRT) (Barr et al. 2013) to determine the significance of the full model (Forstmeier and Schielzeth 2011). For the significant full model, the slope estimates of all substances were extracted from the model results. To determine the substances whose levels changed most with swelling stage, we compared slope estimates to the average slope estimate and focused on those substances whose absolute slope estimate was higher than the average absolute slope estimate + 2SDs (Weiß et al. 2018).

## Results

Chemical profiles of females at the same swelling stage were significantly more similar than profiles of females at different swelling stages (ANOSIM, *R* = 0.07, *P* = 0.026), although the effect was rather small when whole profiles were considered.

**Fig. 1:**
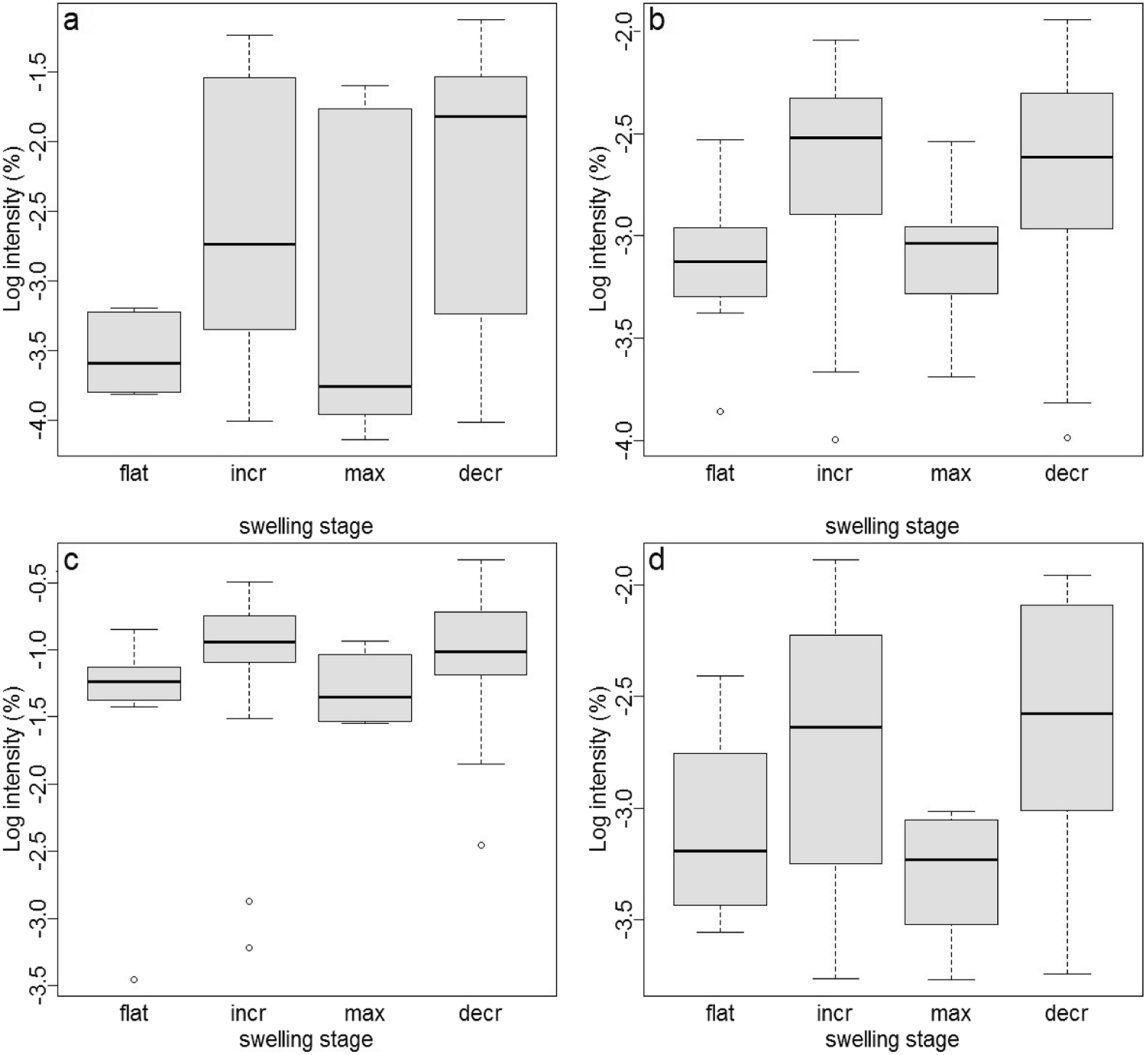
**a–d** Log-transformed intensity (%) of the four substances most influenced by swelling stage (see also Table 1). Boxplots show medians and first and third quartiles. Retention times (RTs) and standardized peak area means ± SD are as follows: **a** RT 42.40, 1.97 ± 2.46; **b** RT 43.49, 0.48 ± 0.43; **c** RT 44.39, 13.71 ± 8.45; **d** RT 48.46, 0.55 ± 0.56

The chemical composition of substances with log-transformed abundances was affected by swelling stage (LRT, *χ*^2^ = 10.77, *P* = 0.013). Thus, the relation of these affected substances to the rest of the respective odor profile changed depending on the swelling stage. This effect was most pronounced for four substances (i.e., their absolute slope estimate was higher than the absolute slope estimate + 2SDs). These substances were all tentatively identified as steroids (Table 1), and were least abundant in samples collected during the maximal swelling as well as the flat phase, and most abundant during the increase and decrease phases of swelling (Fig. 1).

**Table 1:** Substances most affected by swelling stage with* log-transformed* abundances, including retention times (*RTs*), tentative identification [National Institute of Standards and Technologies (NIST) Mass Spectral Library; NIST 14] with probability (best hit) or structural classification, substance class and the largest differences between the absolute slope estimates of swelling stages (average absolute slope estimate ± SD, 0.096 ± 0.08)

RTs	Best library hit/structural classification	Probability	Substance class	Slope estimates
42.40	Unknown steroid (cholestadiene-like)	–	Steroid	0.59
43.49	Cholesta-2,4-diene	94	Steroid	0.32
44.39	Cholesta-3,5-diene	94	Steroid	0.38
48.46	Cholesta-5-en-3-ol (3.beta.)-, acetate	98	Steroid	0.38

For more details, see Fig. 1 and supplementary Online Resource Table 2; for mass spectra, see supplementary Online Resource Fig. 2

**Fig. 2:**
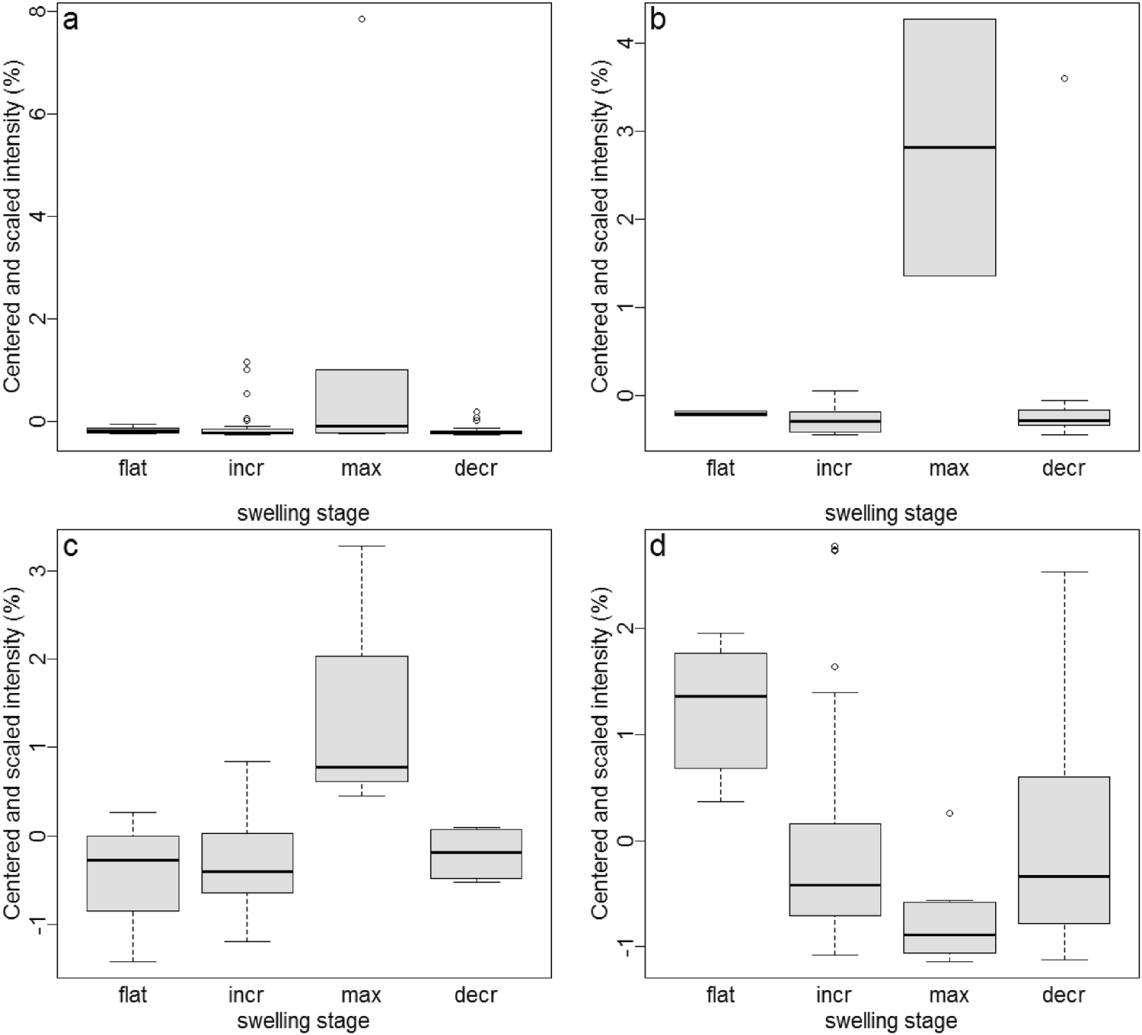
**a–d** Centered and scaled intensity (%) of the four substances most influenced by swelling stage (see also Table 2). Boxplots show medians and first and third quartiles. RTs and standardized peak area means ± SD are as follows: **a** RT 32.26, 0.26 ± 1.05; **b** RT 34.65, 0.12 ± 0.25; **c** RT 40.36, 0.03 ± 0.02; **d** RT 48.80, 0.35 ± 0.31

For substances with centered and scaled abundances, chemical composition was also affected by swelling stage (LRT, *χ*^2^ = 19.28, *P* < 0.001). In these cases, the substance-specific abundances changed between the profiles of different swelling stages. The swelling stage effect was most pronounced for four of the substances (i.e., their absolute slope estimate was higher than the absolute slope estimate + 2SDs), which were different from the most affected substances of the log-transformed model. One of these substances was tentatively identified by the library search as a wax ester (Fig. 2a), whereas two substances had spectra with *m/z* patterns typical of long-chain alkylic structures (Fig. 2b, d), and one substance was thought to feature a phenyl substructure (Fig. 2c) based on its structural classification (see Table 2). Three of the substances were most abundant in samples collected during maximal swelling compared to the other swelling stages, while one of the substances with an alkylic (sub)structure was most abundant during the flat phase and least abundant in samples collected during maximal swelling (see Fig. 2).

**Table 2:** Substances most affected by swelling stages with* centered* and* scaled* abundances, including RTs, tentative identification (NIST 14 library) with probability (best hit) or structural classification, substance class and the largest differences between the absolute slope estimates of swelling stages (average absolute slope estimate ± SD, 0.273 ± 0.20)

RT	Best library hit/structural classification	Probability	Substance class	Slope estimates
32.26	Dodecanoic acid, isooctyl ester	91	Ester	0.87
34.65	Unknown [long-chain alkylic (sub)structure]	–	Unknown	0.91
40.36	Unknown phenol	–	Phenol	0.67
48.80	Unknown [long-chain alkylic (sub)structure]	–	Unknown	0.86

For more details, see Fig. 2 as well as supplementary Online Resource Table 2; for mass spectra, see supplementary Online Resource Fig. 2. For abbreviations, see Table 1

## Discussion

The results of the present study support the presumption that olfaction is related to reproduction in chimpanzees, as the chemical profiles of the female chimpanzees varied significantly with sexual swelling stage. Initial evidence was provided by the slightly greater similarities of the whole chemical profiles of samples taken during the same swelling stage compared to those taken at different swelling stages. These findings were corroborated by a significant change in chemical composition across swelling stages that was most pronounced with respect to eight substances. Thus, the results reveal that olfactory changes exist that mirror changes in fertility at least as closely as the visual signal of sexual swelling. However, our methodological approach did not allow us to address whether olfactory cues provide more precise information which could allow males to pinpoint the time of ovulation more precisely. Hence, further studies that include hormonal measurements are needed to unravel the temporal dynamics of olfactory cues in the context of reproduction.

The effect of swelling stage on chemical composition was tested with two different approaches. Peak areas were either log transformed or centered and scaled when used in the models. In the case of log-transformed peak areas, affected substances differ in contrast to the remaining chemical profile. Hence, information on the state of fertility seems to be provided by the status quo of the chemical composition, and comparisons over time are thus not necessary. When peak areas are centered and scaled, the analysis is focused on differences between, instead of within, the samples (van den Berg et al. 2006). Thus, particular substances that are found to be affected when peak areas are centered and scaled differ in their levels between samples (i.e., over time or swelling stages). In this scenario, it seems to be the variation (or differences) over the course of the menstrual cycle that provides information on fertility stage. A male would thus need to regularly check a female’s odor over time to unravel this information. As expected, the two approaches revealed different chemical substances that were most affected by swelling stage.

Four chemical substances were found to be affected by swelling stage when abundances were log-transformed. All of them were cholesterol derived, and were more abundant during the increasing and decreasing swelling stages compared to the maximal swelling and flat stage. Cholesterol-derived substances are ubiquitous in the chemical profiles of mammals (Charpentier et al. 2012) and are precursors for smaller molecules produced by bacterial degradation (Ezenwa and Williams 2014). Specifically, cholesterol is the precursor for steroid hormones such as estradiol and progesterone (Hu et al. 2010). Swelling of the anogenital skin is estrogen dependent, and detumescence occurs after a rise in progesterone, which inhibits the effects of estrogen (Gillman and Stein 1941; Graham et al. 1972; Emery and Whitten 2003). Tumescence of the sexual swelling occurs due to the intracellular accumulation of water in the anogenital tissue, which is lost during detumescence (Krohn and Zuckerman 1937; Clarke 1940). The consistent pattern of the four most affected substances, i.e., that their levels were highest during tumescence and detumescence, leads to the assumption that these chemical changes arise due to physiological changes that occur during swelling. This chemical information being constistent with the visual signal of increasing or decreasing swelling, could enable males to recognize the beginning and end of the female fertile period, allowing them to focus their monopolization and mating efforts on females which are most fertile.

Moreover, four substances with centered and scaled abundances were found to be related to swelling stage. Similar types of substances, such as long-chain fatty acid esters, substances with alkylic structures, and phenols, were previously detected, e.g., in skin emanations [humans (Bernier et al. 2000)] and in genital secretions [*L. catta* (with the exception of phenols) Boulet et al. 2009]. These four substances were related to the maximal swelling stage, as three of them were most abundant and the other one was least abundant during this phase compared to the other swelling stages. Overall, our results suggest that the chemical composition of female chimpanzee odor changes over the menstrual cycle in accordance with sexual swelling stage. Thus, body odor can be considered at least as a proxy of fertility similar to swelling stage, which has been well established as an imprecise fertility signal.

An olfactory trait related to reproduction is expected to be present consistently within the chemical profile of female odor to allow males to infer female fertility from it. Thus, we checked whether the most affected substances as shown by the two models were continuously present across all the females and swelling stages. All of the substances were found for all six females as well as at all of the swelling stages, and most of them were present in at least 80% of the samples for at least two swelling stages. However, two of the substances indicated by the centered and scaled model (an alkylic compound with an RT of 34.65 min and an unknown phenol with an RT of 40.36 min) were only present in a  maximum of 43% of samples per swelling stage (maximal swelling and increasing/decreasing swelling stages). If substances are not always present in the chemical profile of female odor, they are not very suitable for use by males as reliable indicators of female reproductive state. Future studies that include a higher number of samples of female odor will help researchers to examine this subject in more detail.

While our results indicate that body odor does indeed change over a female’s reproductive cycle, our methodology did not allow us to assess whether olfactory changes are as precise as the visual signal, or potentially even more precise. In fact, in the context of an evolutionary arms race, we would expect males to strive to pinpoint the exact timing of ovulation by using a fertility trait, and thus that they would benefit from an olfactory cue that is more precise than the visual cue of sexual swelling. Females, on the other hand, would profit from generally confusing paternity by providing the rather imprecise visual signal of fertility which could be strengthened by an additional, imprecise olfactory cue. However, high-ranking male chimpanzees in particular have been observed to start copulating only in the last 3–4 days of maximal swelling (Deschner et al. 2004). As the duration of maximal swelling is variable and its termination cannot be deduced according to the point at which it starts, such observations suggest that high-ranking male chimpanzees in particular, which are able to approach females to within a very close distance, may rely on additional and more precise indicators of ovulation, i.e., presumably olfactory traits. In humans, odor has been proposed as a more precise indicator for (approaching) ovulation than visual signs (Singh and Bronstad 2001; Havliček et al. 2006; Haselton and Gildersleeve 2011; Gildersleeve et al. 2012). Relying on more than one of the senses for the inspection of a female is likely to be advantageous for male chimpanzees too. The visual signal could give initial information about the state of fertility of a female at short/medium distance, which may then especially attract high-ranking males with priority of access, allowing them to undertake an olfactory inspection at close proximity to gain additional information (Matsumoto-Oda et al. 2007). Such olfactory information could potentially be non-redundant to the visual information (see also Higham and Hebets 2013), but our study design did not allow us to determine whether the olfactory cue is redundant or not.

Thus, an important next step building on our current results, that show that chemical composition changes over the menstrual cycle in accordance with stages of sexual swelling, would be  investigating whether chemical profiles indeed provide more precise information than sexual swellings. To investigate this in more detail, analyses of cycle-related hormones are needed to determine the exact timing of ovulation, which could not be accomplished at the time that this study was conducted. This could be undertaken by using non-invasive hormonal measurements, as applied in previous studies to assess the timing of ovulation in non-human primates (Deschner et al. 2004; Engelhardt et al. 2006; Dubuc et al. 2012; Young et al. 2013; Douglas et al. 2016).

Moreover, recently adapted sampling techniques now allow a wider range of chemical substances to be sampled (Kücklich et al. 2017). The traditionally used cotton swab, which was also used in this study, primarily captures semi- to non-volatile substances (Birkemeyer et al. 2016). These types of larger molecules are then degraded by skin bacteria into smaller, volatile molecules that constitute the odor profile of an individual (Ezenwa and Williams 2014) that can be perceived by the main olfactory system (Dulac and Torello 2003). Future studies will certainly benefit from these recent methodological developments for the direct sampling of volatile substances, and for the correlation of their abundances to individual traits of animals.

In conclusion, this study adds much-needed information on the role of olfaction in great apes by showing that the chemical composition of body odor is associated with a visual signal of fertility in chimpanzees. Our results indicate that olfactory cues related to reproduction exist in a great ape species with visual fertility advertisement, and provide a valuable basis for further research on more specific questions on reproduction in chimpanzees and olfaction in great apes in general. In fission–fusion societies, where group composition varies and visual information is short lived, olfaction may allow males to fine-tune their sociosexual behavior and to optimize their reproductive investment.

## Supplementary Information

Below is the link to the electronic supplementary material.

Supplementary file1 (DOCX 658 KB)

## Data Availability

The dataset generated and analyzed during the current study is available on reasonable request.
