# Supplementary material for: Towards an understanding of multimodal traits of female reproduction in chimpanzees
Source: Primates. 2022 Jun 28;63(4):365–76. doi: 10.1007/s10329-022-00995-1 (PMC9273533; doi:10.1007/s10329-022-00995-1)
Supplement: Supplementary file 1 — Supplementary file1 (DOCX 658 KB) [file 10329_2022_995_MOESM1_ESM.pdf]

ONLINE RESOURCE

Additional Methods and Results

Housing Conditions of the Chimpanzees

The Leipzig Zoo provides indoor and outdoor enclosures accessible depending on weather conditions. The chimpanzees have access to water *ad libitum* and are fed at least four times a day with fresh fruits, vegetables, leaves, herbs, seeds, pellets and branches. Once per week the apes are fed with cooked meat and eggs. Additionally, they are provided with enrichment objects (hidden or special food items) on a daily basis.

For the study analyzing chemical compositions of skin exudates, we took samples from six female chimpanzees. The individuals were housed in two different social groups (Group 1 & 2). One female (ID 1) lived in a group consisting of 16 individuals (six males, ten females, Group 1) and had given birth to two infants, five and two years prior to sample collection, but none of the infants had survived. The other females were housed together with a sterilized adult male and all of them were cycling regularly but were nulliparous (Group 2).

Online Resource Table 1.

Females from the two social groups sampled for chemical analysis. Females belonged to two different social groups and were all regularly cycling, not lactating and received no contraception. Given are also number of samples per female (total and in the four respective swelling stages).

Subject	Group	Age in 2013 (at study begin)	Number of samples (total)	Number of samples (detumescent)	Number of samples (increasing)	Number of samples (maximal)	Number of samples (decreasing)
ID 1	1	17	15	4	2	5	4
ID 2	2	19	15	10	2	3	0
ID 3	2	19	14	5	0	8	1
ID 4	2	20	20	12	0	8	0
ID 5	2	13	15	7	2	6	0
ID 6	2	13	18	5	1	10	2

Online Resource Table 2.

Tentative identification (accepted NIST08 library hit) or structural classification of all relevant substances with retention time (RT), probability (best hits), boiling point (predicted data from www.chemspider.com 2015), substance class and references (reported from other species) are given.

Substance	Identification (accepted library hit)	RT	Prob.	Boiling point (at 760 mmHg)	Classification	References
RT026	Dodecanoic acid, isooctyl ester	32.26	91	354.6 ±10.0 °C	ester	*Dodecanoic acid* in Zidat et al. 2018: anal scent gland secretion of Alpine marmot; Rosell and Sundsdal 2001: anal scent secretion of Eurasian beaver; Bernier et al. 2000: skin emanations from humans; *fatty acids* in Boulet et al. 2009: secretions from ring-tailed lemurs
RT036	Unknown (long-chain alkylic (sub)structure)	34.65	---	---	unknown	*Substances with alkylic structures* in Bernier et al. 2000: skin emanations from humans; Boulet et al. 2009: secretions from ring-tailed lemurs
RT075	Unknown phenol	40.36	---	---	aromatic hydrocarbon	*Phenols* in Bernier et al. 2000: skin emanations from humans
RT089	Unknown steroid (cholestadiene-like)	42.40	---	---	steroid	Charpentier et al. 2012: review
RT101	Cholesta-2,4-diene	43.49	94	451.3 ±12.0 °C	steroid	Zidat et al. 2018: anal scent gland secretion of Alpine marmot; Harris et al. 2014: spur secretions of short-beaked echidna
RT110	Cholesta-3,5-diene	44.39	94	458 ±12.0 °C	steroid	Bernier et al. 2000: skin emanations from humans; Costello et al. 2014: skin secretions from humans; Harris et al. 2014: spur secretions of short-beaked echidna
RT138	Cholest-5-en-3-ol (3.beta.)-, acetate	48.46	98	493.3 ±24.0 °C	steroid	Helmy et al. 2019: blood samples of rats
RT139	Unknown (long-chain alkylic (sub)structure)	48.80	---	---	unknown	*Substances with alkylic structures* in Bernier et al. 2000: skin emanations from humans; Boulet et al. 2009: secretions from ring-tailed lemurs

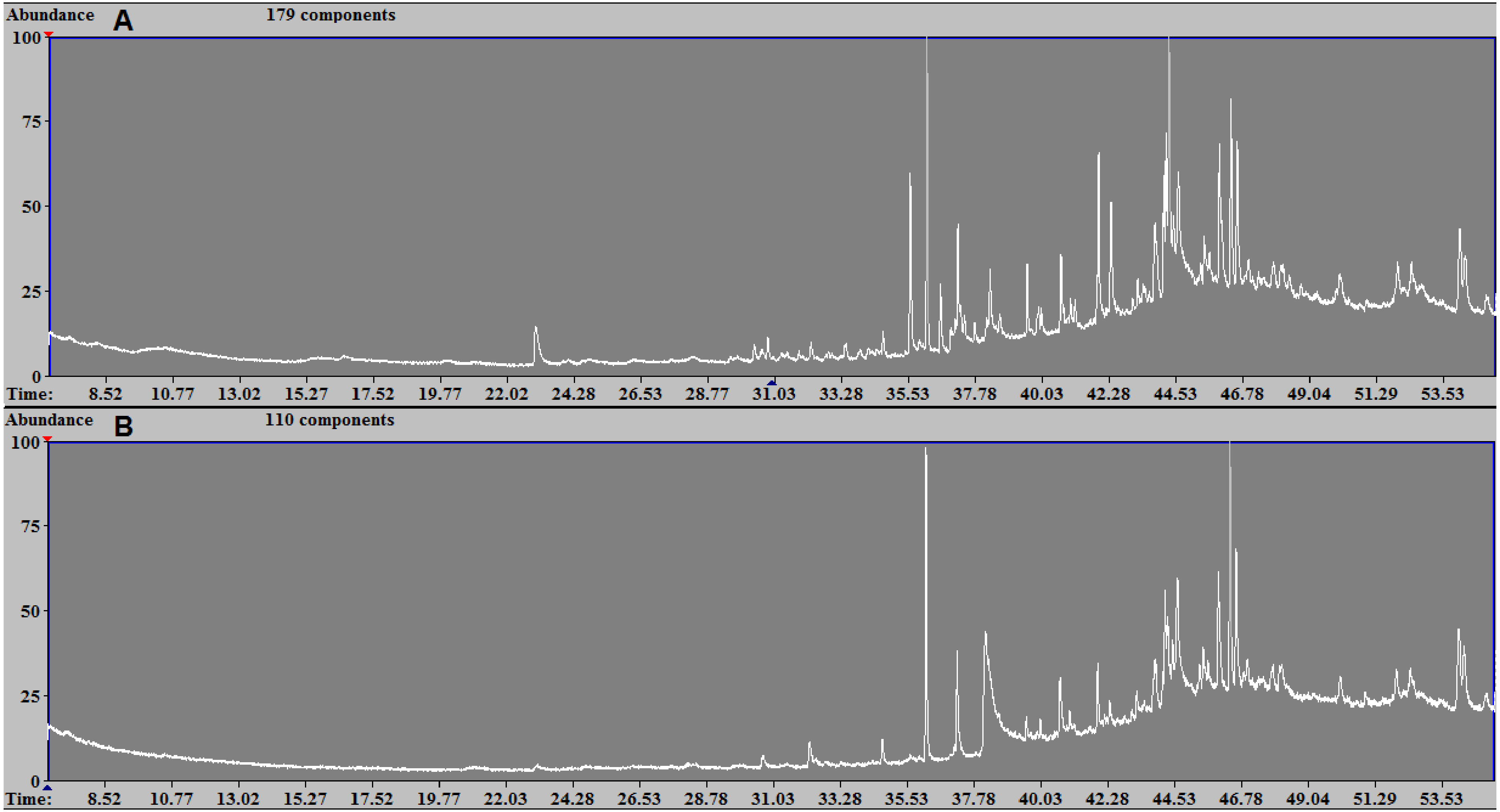

Online Resource Figure 1.

Example chromatograms from an animal sample (A) and a blank sample (B).

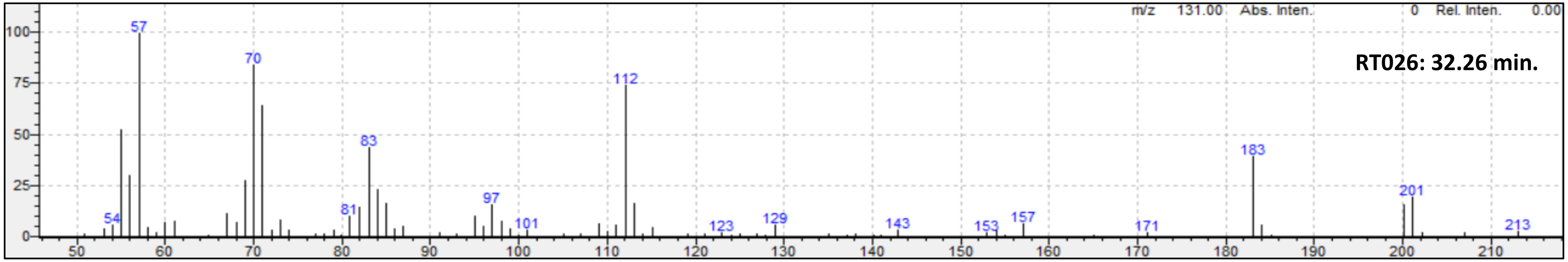
m/z 131.00 Abs. Inten. 0 Rel. Inten. 0.00
RT026: 32.26 min.
57
70
112
83
183
201
97
81
54
101
123
129
143
153
157
171
213

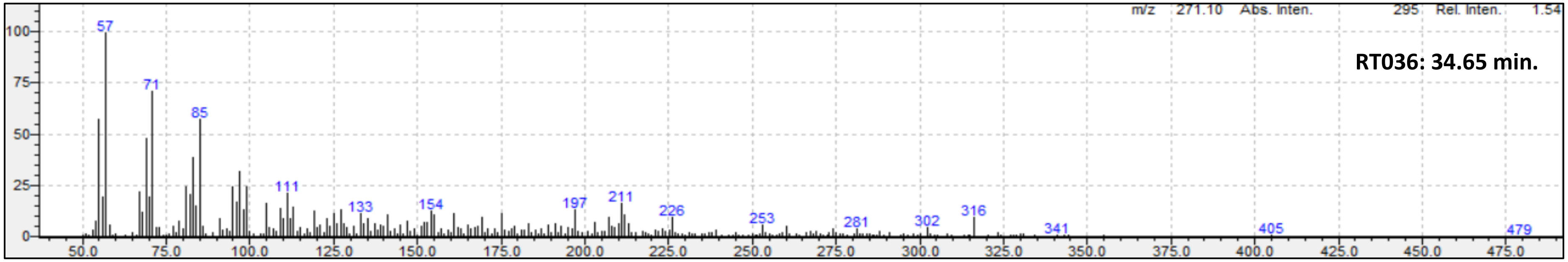
m/z 271.10 Abs. Inten. 295 Rel. Inten. 1.54
RT036: 34.65 min.
57
71
85
111
133
154
197
211
226
253
281
302
316
341
405
479

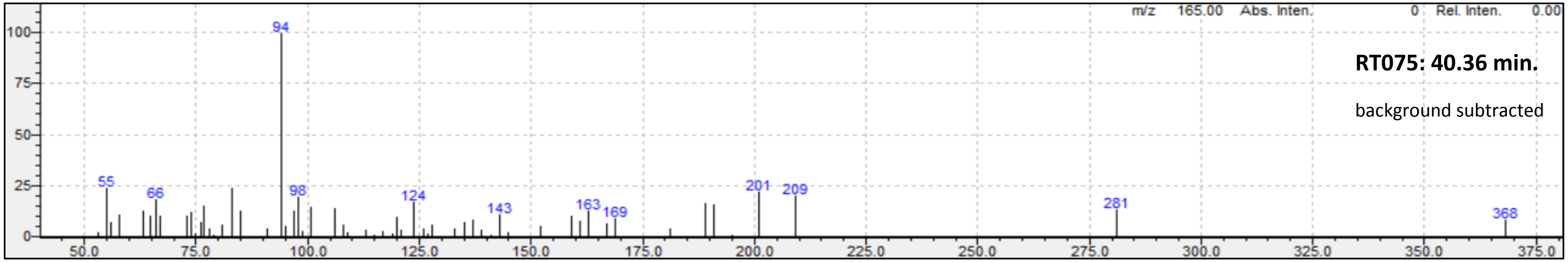
m/z 165.00 Abs. Inten. 0 Rel. Inten. 0.00
RT075: 40.36 min.
background subtracted
94
55
66
98
124
143
163
169
201
209
281
368

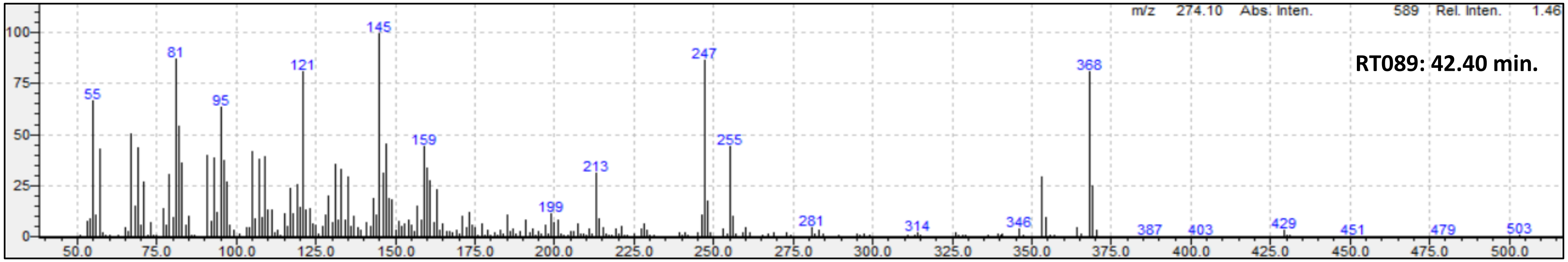
m/z 274.10 Abs. Inten. 589 Rel. Inten. 1.46
RT089: 42.40 min.
145
81
121
55
95
247
368
159
255
213
199
281
314
346
387
403
429
451
479
503

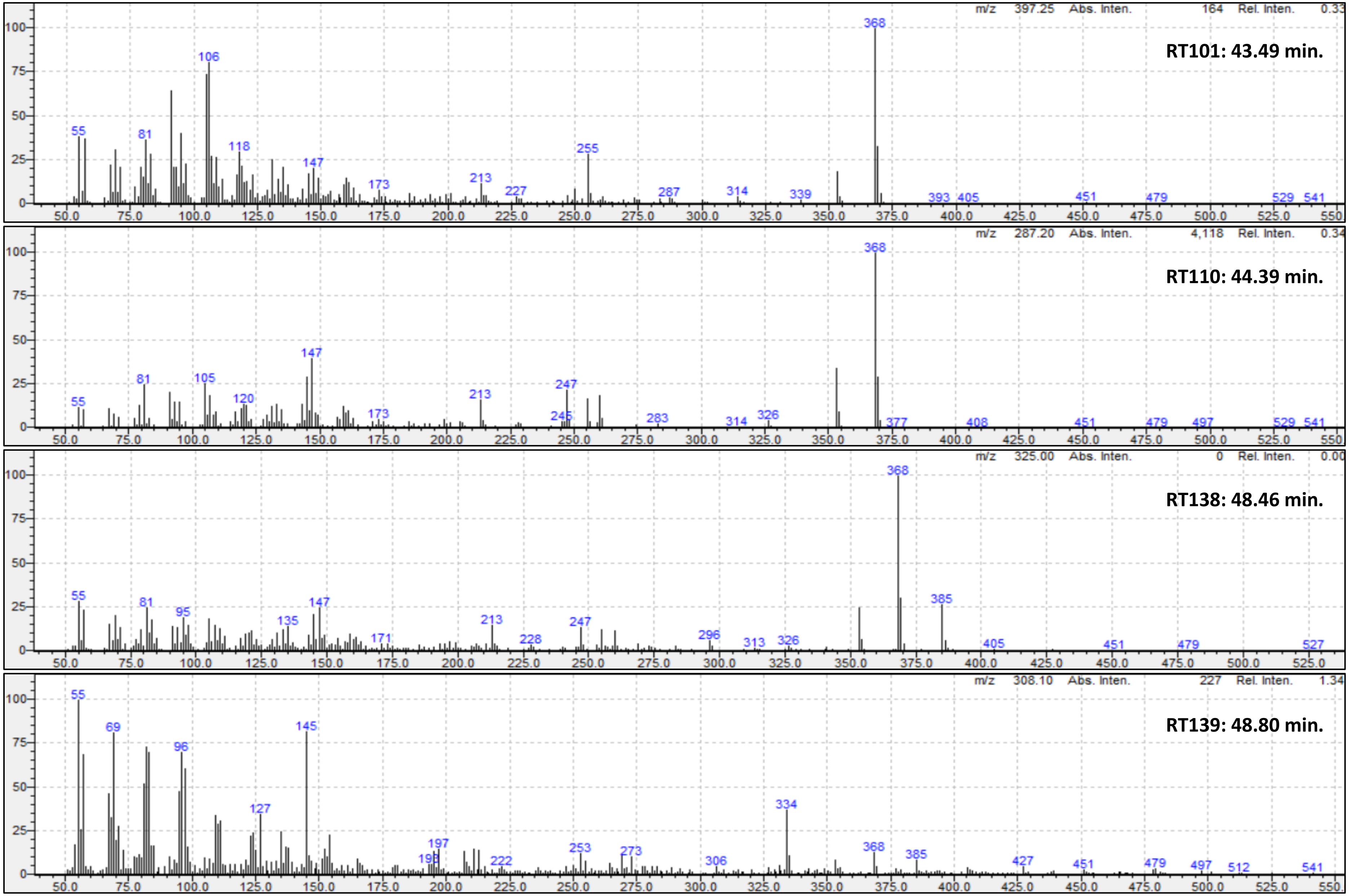

Online Resource Figure 2. Mass spectra of most intense peaks from substances found to be most affected.

References

Bernier, U.R., Kline, D.L., Barnard, D.R., Schreck, C.E., and Yost, R.A. 2000. Analysis of Human Skin Emanations by Gas Chromatography/Mass Spectrometry. 2. Identification of Volatile Compounds That Are Candidate Attractants for the Yellow Fever Mosquito (*Aedes aegypti*). Anal Chem. 72:747–756.

Boulet, M., Charpentier, M., and Drea, C. 2009. Decoding an olfactory mechanism of kin recognition and inbreeding avoidance in a primate. BMC Evol Biol. 9:281.

Charpentier, M.J.E., Barthes, N., Proffit, M., Bessière, J.-M., and Grison, C. 2012. Critical thinking in the chemical ecology of mammalian communication: roadmap for future studies. Funct Ecol. 26:769–774.

Costello, B. de L., Amann, A., Al-Kateb, H., Flynn, C., Filipiak, W., Khalid, T., Osborne, D., and Ratcliffe, N.M. 2014. A review of the volatiles from the healthy human body. J Breath Res. 8:014001.

Harris, R.L., Holland, B.R., Cameron, E.Z., Davies, N.W., and Nicol, S.C. 2014. Chemical signals in the echidna: differences between seasons, sexes, individuals and gland types. J Zool. 293:171–180.

Helmy, A., Ghanem, H., Ibrahim, N., Gendy, A.N.E., Hussein, N.S., and Abdel-Hamid, A. 2019. Targeted metabolomics reveals the therapeutic impact of Eclipta prostrata on diet-induced non-alcoholic fatty liver disease in rats. J Appl Pharm Sci. 9:77–90.

Rosell, F., and Sundsdal, L.J. 2001. Odorant Source Used in Eurasian Beaver Territory Marking. J Chem Ecol. 27:2471–2491.

Zidat, T., Dufour, A.-B., Meiffren, G., Gabirot, M., Comte, G., and Allainé, D. 2018. Anal scent gland secretions inform on sexual maturity, sex and social status in the Alpine marmot, *Marmota marmota* (Rodentia: Sciuridae): a role in intrasexual competition in cooperative breeders? Biol J Linn Soc. 125:229–239.
